# Anti-centromere antibodies and associated autoimmune diseases

**DOI:** 10.5339/qmj.2023.sqac.28

**Published:** 2023-11-26

**Authors:** Kaaouch Hanae, Bhallil Ouahiba

**Affiliations:** ^1^Immunology Department, Central Medical Analysis Laboratory, University Hospital Hassan II, Fez Email: hanae.kaaouch@gmail.com; ^2^Faculty of Medicine, Dental and Pharmacy, Sidi Mohamed Ben Abdellah University, Morocco

## Abstract

**Introduction:** Anti-centromere antibodies (ACAs) are a variety of anti-nuclear autoantibodies (ANA) directed against different kinetochore proteins. They are sought on HEp2 substrates by indirect immunofluorescence technique (IFI), where they appear in the form of about forty fine nuclear punctuations attached to the chromosomes during their different cell cycle phases. They are encountered in several autoimmune diseases (AID), frequently in scleroderma. This work aims to retrospectively analyze serum samples containing anti-centromere antibodies and the associated pathologies.

**Materials and methods:** Eleven serum positive for ACAs were studied among 821 requests for ANA analyses sent to the Immunology Department, Central Laboratory for Medical Analyzes, CHU Hassan II-Fez for three years. Two techniques were used: indirect immunofluorescence for the ANA research and ELISA or Immunodot for identifying antigenic specificities according to the suppliers’ recommendations (D-Tek).

**Results:** The mean age of patients was 50 ± 18 years. A female predominance (81.8%) was observed, with a sex ratio F/M of 4.5. The research of Anti-nuclear antibodies was positive in immunofluorescence in all cases. With a centromeric appearance in 9 cases and a speckled appearance in 2 cases, the titer varied between 160 and 1280. Antigenic identification by Immunodot showed that anti-CENP A and CENP B were found in 7 cases, and anti-CENP B was identified in 4 cases. The results by pathology show a significant association of ACAs with the clinical suspicion of systemic sclerosis (45%).

**Conclusion:** The presence of ACAs can be associated with several AIDs, mainly scleroderma. These autoantibodies represent an important diagnostic and prognostic tool by defining immuno-clinical forms of the disease.
